# Nonsteroidal anti‐inflammatory drugs in acute viral respiratory tract infections: An updated systematic review

**DOI:** 10.1002/prp2.925

**Published:** 2022-02-26

**Authors:** Nima Azh, Farzaneh Barzkar, Nogol Motamed‐Gorji, Parmida Pourvali‐Talatappeh, Yousef Moradi, Roya Vesal Azad, Mitra Ranjbar, Hamid Reza Baradaran

**Affiliations:** ^1^ School of Medicine Iran University of Medical Sciences Tehran Iran; ^2^ Center for Educational Research in Medical Sciences Iran University of Medical Sciences Tehran Iran; ^3^ Social Determinants of Health Research Center Research Institute for Health Development Kurdistan University of Medical Sciences Sanandaj Iran; ^4^ School of Public Health Iran University of Medical Sciences Tehran Iran; ^5^ Department of Infectious Diseases School of Medicine Iran University of Medical Science Tehran Iran; ^6^ Ageing Clinical and Experimental Research Team Institute of Applied Health Sciences School of Medicine Medical Sciences Nutrition University of Aberdeen Aberdeen UK; ^7^ Department of Epidemiology School of Public Health Iran University of Medical Sciences Tehran Iran

**Keywords:** acute respiratory distress syndrome, acute respiratory tract infections, coronavirus disease 2019, nonsteroidal anti‐inflammatory drugs, systematic review

## Abstract

In this systematic review, we aimed to assess the efficacy and safety of nonsteroidal anti‐inflammatory drugs (NSAIDs) in treating respiratory tract infections in adults and children. PubMed, Scopus, Web of Science, Cochrane, and Embase databases were searched. A total of 34 randomized clinical trials were included in this systematic review. We assessed the risk of bias of all included studies using the Cochrane tool for risk of bias assessment. The evidence on ibuprofen, naproxen, aspirin, diclofenac, and other NSAIDs were rated for degree of uncertainty for each of the study outcomes and summarized using the grading of recommendations assessment, development, and evaluation (GRADE) approach. Our findings suggest that high‐quality evidence supports the use of NSAIDs to reduce fever in both adults and children. However, the evidence was uncertain for the use of NSAIDs to reduce cough. Most studies showed that NSAIDs significantly relieved sore throat. The evidence for mortality and oxygenation is limited. Regarding the adverse events, gastrointestinal discomfort was more frequently reported in children. For adults, our overall certainty in effect estimates was low and the increase in gastrointestinal adverse events was not clinically significant. In conclusion, NSAIDs seem to be beneficial in the outpatient management of fever and sore throat in adults and children. Although the evidence does not support their use to decrease mortality nor improve oxygenation in inpatient settings, the use of NSAIDs did not increase the rate of death or the need for ventilation in patients with respiratory tract infections. Further studies with a robust methodology and larger sample sizes are recommended.

## Graphical abstract

A visual summary of efficacy and safety of NSAIDs in acute viral respiratory tract infections.

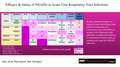

## 1 INTRODUCTION

Acute viral respiratory tract infection (ARTI) is the most common condition in humans regardless of age.^1^ These infections are mostly self‐limiting and do not require antimicrobial therapy, and patients are thus treated based on their presenting symptoms. This wide range of symptoms can be caused by a systemic inflammatory response as well as localized respiratory tract involvement in both the upper and lower sections.^2^

Nonsteroid anti‐inflammatory drugs (NSAIDs) are the most commonly used medications for relieving signs and symptoms of ARTI.^3^ NSAIDs suppress inflammation through inhibition of cyclooxygenase, which in turn, decrease the production of prostaglandins and thromboxanes.^4^, ^5^, ^6^

The coronavirus disease 2019 (Covid‐19) is a viral respiratory tract infection that can cause a wide range of symptoms involving the upper and lower respiratory systems. Also, since most severe symptoms in this new disease stem from uncontrolled inflammation, medications with anti‐inflammatory activity were proposed as potential disease‐modifiers. NSAIDs are among the most commonly prescribed medications in this novel respiratory infection. However, many experts are concerned about the safety and efficacy of this class of medications in Covid‐19.

Several studies and systematic reviews have been performed to demonstrate the efficacy of NSAIDs in alleviating ARTI symptoms.^3^ However, no consensus has been reached on this issue; therefore, we aimed to comprehensively synthesize the evidence for the efficacy and safety of different NSAIDs in acute viral respiratory tract infections as an update to these previously performed systematic reviews.

## 2 MATERIALS AND METHODS

### 2.1 Search strategy

PubMed, Embase, Scopus, Web of Science, and Cochrane Library databases were searched with search syntaxes designed by a medical librarian (R.V.A.), and a team of physicians and clinical epidemiologists. The search terms and syntax are shown in Appendix 1. The literature review was conducted in November 2020. The results were screened by independent authors (N.A., F.B., N.M‐G., and P.P.). Relevant articles were then screened by abstract and full‐text. An independent investigator (N.A.) double‐checked every entry before submission in the final data sheet and resolved arguments through online discussions. We quantified graph data using Getdata Graph Digitizer for graphs without available values.^8^ Finally, 34 studies were included in the systematic review. This study is reported following the PRISMA (Preferred Reporting Items for Systematic Reviews and Meta‐Analyses) recommendations.^7^

### 2.2 Inclusion and exclusion criteria

We included published randomized clinical trials assessing the clinical effectiveness and adverse effects of NSAIDs (including aspirin, naproxen, ibuprofen, diclofenac) compared with placebo or other medications in all dosages and forms as the main treatment of acute viral respiratory tract infections in adults and children.

Studies evaluating chronic respiratory conditions, nonviral etiology of ARTI, central hyperthermia, and infantile acute respiratory distress syndrome (ARDS) were excluded. Unobtainable non‐English full‐texts were excluded. Also, studies conducted before 1980 were excluded because of the lack of statistical and methodological details required for evidence synthesis.

### 2.3 Data extraction

Our outcomes included cough, sore throat, fever, gastrointestinal adverse events, as well as length of stay (LOS), development of ARDS and need for ventilator support, and mortality. Data were extracted for each of the included studies by two independent authors and recorded in uniform Excel forms.

### 2.4 Quality assessment

We assessed the quality of included randomized controlled trials (RCTs) using the Cochrane tool for risk of bias assessment.^9^ Risk of bias assessment was performed by two authors independently for every included study, and arguments were solved through online discussion sessions (N.A., F.B., N.M‐G., and P.P. assessed the risk of bias). We used the risk of bias visualization (ROBVIS) tool to create traffic light plots and bar plots.^10^

To assess the overall certainty of the evidence for every outcome in the summary of findings table, we employed The grading of recommendations assessment, development, and Evaluation (GRADE).^11^ In this approach, the obtained estimate for each outcome is rated for the degree of confidence (certainty) based on the risk of bias, directness, precision, consistency, publication bias, the magnitude of effect, dose‐response gradient, and plausible confounding factors. Accordingly, the effect estimates are described to have a high, moderate, low, or very low degrees of certainty. GRADEpro GDT online software was used to perform the GRADE approach and to create a summary of findings tables.^12^

### 2.5 Statistical reporting and meta‐analysis

We grouped the studies evaluating the same drugs together and estimated effect estimates for outcomes of individual studies. The effect estimates of dichotomous outcomes were expressed as risk ratios (RR) with 95% CIs and continuous data was expressed as mean differences (MDs) or standardized mean differences with 95% CI. The median, first, and third quartiles were reported for ordinal outcomes.

Because of the heterogeneity of the reported scales, diversity of intervention agents, dose and duration, population age group, and contexts, we were only able to perform a meta‐analysis on the gastrointestinal adverse events of aspirin. Therefore, we narratively summarized the rest of the outcomes in GRADE tables.

## 3 RESULTS

### 3.1 Study inclusion and characteristics

**FIGURE 1:**
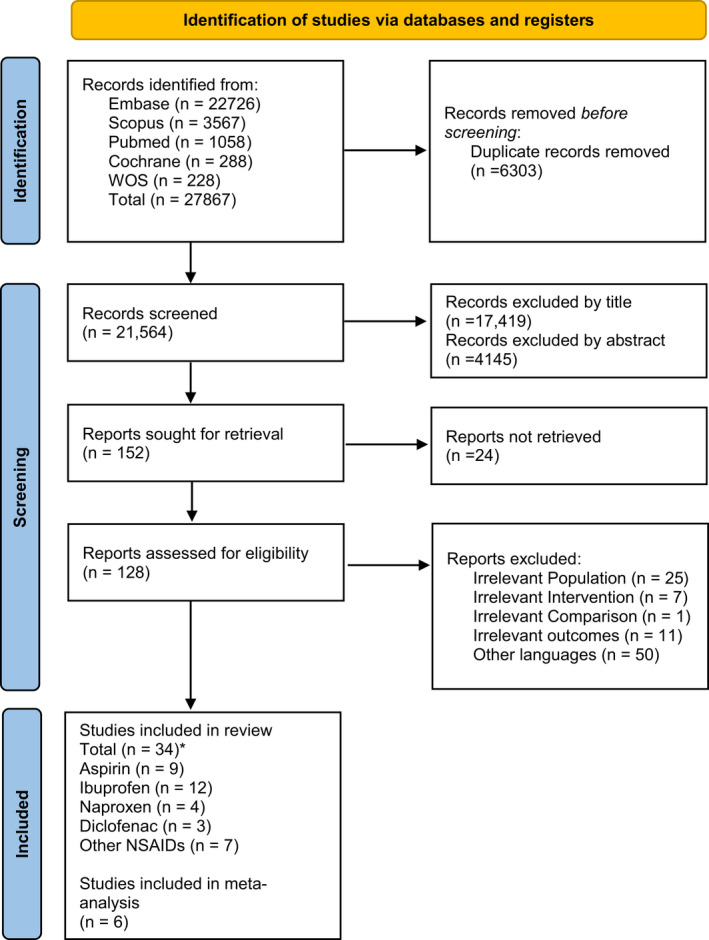
Non‐steroidal anti‐inflammatory drugs in Acute Viral Respiratory tract infections and Acute Respiratory Distress Syndrome (ARDS) *An Article by Weckx et al. is included in both Diclofenac and Other NSADs groups. *From:* Page MJ, McKenzie JE, Bossuyt PM, Boutron I, Hoffmann TC, Mulrow CD, et al. The PRISMA 2020 statement: an updated guideline for reporting systematic reviews. BMJ 2021;372:n71. 10.1136/bmj.n71

Initial search retrieved 27 867 articles across 5 databases, of which 6303 were duplicates and 152 were considered for full‐text screening. Finally, 34 studies (9 for aspirin, 4 for naproxen, 12 for ibuprofen, 3 for diclofenac, and 6 for other NSAIDs) were included in our systematic review. Figure 1 presents a detailed summary of the search process based on the PRISMA guideline.

**TABLE 1:** Study characteristics

NSAID	Study	Participants age & setting	Intervention (number of participants)	Comparison (number of participants)	Outcomes								
					Fever	Cough	Sore Throat		ARDS		Length of Stay / Mortality		Gastrointestinal Adverse Events
**Ibuprofen**	Llor, C. 2013 Spain	Adult Inpatient ARTI	Ibuprofen 600 mg TDS For 10 days (n = 136)	Placebo (n = 143)		Number of days with cough Mean difference (95% CI^d^)							GI upset RR (95% CI)

2 1.5 to 2.5	1.84 0.55 to 6.15

Bernard, G. R. 1997 North America	Adult Inpatient Sepsis Criteria	Ibuprofen IV/inf./ 10 mg/kg QID (maximal dose: 800 mg daily) for 2 days (n = 224)	Placebo (n = 231)	4 h Fever Mean difference (95% CI)				24 h Minute‐Ventilation (L/min) Mean difference (95% CI)		30‐day Mortality RR (95% CI)		GI Bleeding RR (95% CI)

−0.80 −0.78 to −0.82	0.92 0.85 to 0.99		0.88 0.70 to 1.11		0.58 0.26 to 1.29

Bouroubi, A. 2017 Europe	Adult Outpatient Sore Throat	Ibuprofen 25mg lozenge p.o. up to 6 doses daily for 4 days (n = 194)	Placebo lozenges p.o. for 4 days (n = 191)			4th day pain Relieved RR (95% CI)						GI upset RR (95% CI)

1.51 1.06 to 2.15		2.95 0.60 to 14.45

Little, P. 2013 United Kingdom	Adult & Pediatrics Outpatient ARTI	Ibuprofen 200–400 mg p.o. QID for 3 days (n = 150)	Paracetamol 15 mg/kg p.o. QID for 3 days (n = 152)									GI upset RR (95% CI)

0.83 0.63 to 1.10

Winther, B. 2001 Denmark	Adult Outpatient URTI	Ibuprofen 400 mg p.o. TDS for 3 days (n = 38)	Placebo (n = 42)	Chilliness improvement 3‐day (0–9 score) Mean difference (95% CI)	Cough improvement 3‐day (0–9 score) Mean difference (95% CI)	Sore Throat improvement 3‐day (0–9 score) Mean difference (95% CI)						

0.63 0.75 to 0.51	0.83 1.05 to 0.61	0.31 0.48 to 0.14	

Gwaltney, J. M., Jr. 2002 USA	Adult Outpatient Rhinovirus Inoculation	Intranasal placebo + oral chlorpheniramine (12 mg extended release) and Tab. Ibuprofen 400 mg BD for 5 days (n = 61)	Intranasal and oral placebos (n = 30)		3rd day Cough (0–4 score) Mean difference (95% CI)	3rd day Sore throat (0–4 score) Mean difference (95% CI)						

−0.12 −0.09 to −0.15	−0.24 −0.20 to −0.28	

Sperber, S. J. 1989 USA	Adult Outpatient Rhinovirus Inoculation	Pseudoephedrine HC1 60 mg + Ibuprofen 200 mg p.o. for 5 days (n = 23)	Pseudoephedrine HC1 60 mg + placebo p.o. for 5 days (n = 23)			Sore Throat (0–18 score) Mean difference (95% CI)						GI upset RR (95% CI)

−1 −3.10 to 1.10		0.33 0.01 to 7.78

Kim, C‐K. 2013 South Korea	Pediatric Outpatient URTI	Dexibuprofen 3.5 or 7 mg/kg p.o. single dose (n = 75)	Ibuprofen 5 or 10 mg/kg p.o. single dose (n = 76)	4 h Fever Mean difference (95% CI)								GI upset RR (95% CI)

0.26 −0.0232 to 0.5432	0.98 0.70 to 1.39

Hay AD. 2008 United Kingdom	Pediatric Outpatient Fever	Ibuprofen 10 mg/kg p.o. q6–8hr for 2 days (n = 52)	Paracetamol 15 mg/kg p.o. q4–6hr for 2 days (n = 52)	Time until first fever clearance (minutes) Mean difference (95% CI)						2nd day Recovered child RR (95% CI)		GI upset RR (95% CI)

				28.8 7.68 to 49.92						0.97 0.52 to 1.80	

2nd day temperature Mean difference (95% CI)	5th day Recovered child RR (95% CI)		0.75 0.39 to 1.43

0 −0.34 to 0.34	0.83 0.49 to 1.38	

Yoon, J. S. 2008 South Korea	Pediatric Outpatient URTI	Ibuprofen 10 mg/kg p.o. single dose (n = 85)	Dexibuprofen 5 mg/kg p.o. single dose (n = 86)	6 h Fever Reduction Mean difference (95% CI)								GI upset RR (95% CI)

				−0.1 ‐0.39 to 0.19							

6 h Fever reduced to normal RR (95% CI)	1.01 0.37 to 2.76

1.40 0.96 to 2.03

Ulukol, B. 1999 Turkey	Pediatric Outpatient URTI	Ibuprofen (suspension) 10 mg/kg p.o. TDS + Anti‐Biotics for 5 days (n = 30)	Paracetamol (suspension) 10 mg/kg p.o. TDS + Anti‐Biotics (n = 30)	Fever improved 2nd day RR (95% CI)	Cough improved 5th day RR (95% CI)							

0.60 0.33 to 1.12	0.79 0.60 to 1.06

Bertin, L. 1991 France	Pediatric Outpatient Sore Throat	Ibuprofen 10 mg/kg p.o. TDS + penicillin for 7 days (n = 77)	Placebo + penicillin for 7 days (n = 76)			2nd day pain relief RR (95% CI)						GI upset RR (95% CI)

1.68 1.27 to 2.23		0.99 0.30 to 3.27
**Naproxen**	Hung 2017 Hong Kong	(H3N2) influenza inpatient Adult	clarithromycin 500 mg, naproxen 200mg, and oseltamivir 75 mg BD for 2 days, followed by 3 days of oseltamivir 75 mg BD (n = 107)	oseltamivir 75 mg BD for 5 days (n = 110)					Respiratory support during hospitalization RR (95% CI)		Days of Acute care hospitalization median (1st to 3rd quartile)		
									0.53 0.33 to 0.83		2 (1–3)	3 (2–4)	
									HDU admission required during hospitalization RR (95% CI)		30‐day mortality RR (95% CI)		
									0.51 0.31 to 0.86		0.11 0.01 to 0.89		
									ICU^h^ admission required during hospitalization RR (95% CI)		90‐day mortality RR (95% CI)		
									0.29 0.06 to 1.38		0.19 0.04 to 0.82		
	Sperber, S. J. 1992 USA	Outpatient Adults Viral inoculation with RSV	Naproxen 400 mg or 500 mg p.o. stat followed by 200 mg or 500 mg TDS for 5 days (n = 39)	Placebo (n = 40)	5th day Chilliness (0–4 score) Mean difference (95% CI)	5th day Cough (0–4 score) Mean difference (95% CI)	5th day Sore throat (0–4 score) Mean difference (95% CI)						GI Upset RR (95% CI)
					0.40 −0.11 to 0.91								
					Fever developed (Temp. >37.5) RR (95% CI)	0.8 −0.17 to 1.80	0.5 −0.93 to 1.90						0.53 0.05 to 5.57
					0.14 0.03 to 0.59								

Gwaltney, J. M., Jr. 1992 USA	Outpatient Adults Viral inoculation with RSV	‐Naproxen 500 mg stat and then 250 mg TDS ‐Ipratropium 80 *mcg (*2 puffs/ nostril) TDS ‐IFN‐a2b 3 million units (0.1 mL/nostril) TDS for 4 days (n = 16)	Placebo (n = 8)									GI Upset RR (95% CI)

1.59 0.07 to 35.15

Salmon Rodriguez, L. E. 1993 Mexico	(Probably inpatient) Pediatrics Sore Throat	Naproxen TDS p.o. for 8 days Dosing: (25 mg per ml): −2.5 ml for 1 to 3 years. −5 ml for 4 to7 years. −7.5 ml for 8 to 10 years. (n = 50)	Nimesulide BD p.o. for 8 days Dosing: (l0 mg per ml): ‐ 2.5 ml for 1 to 3 years. ‐ 5 ml for 4to 7 years. ‐ 7.5 ml for 8 to 10 years. ‐ 10 ml for more than 10 years. (n = 49)									GI Upset RR (95% CI)

2.73 0.95 to 7.89
**Diclofenac**	Grebe 2003 Germany	Adult Outpatient Influenza‐like symptoms	Diclofenac 25mg p.o. daily For 3 days (n = 121)	Placebo (n = 115)	6 h Fever under 37.8˚C RR (95% CI)								GI Upset RR (95% CI)
					2.73 1.27 to 5.86								0.48 0.043 to 5.17
	Weckx 2002 Brazil/ Columbia/ Mexico	Adult Outpatient Influenza‐like symptoms	Diclofenac 75 mg p.o. BD for 5 days (n = 101)	celecoxib 200 mg p.o. BD for 5 days (n = 107)			3rd day Visual Analogue Scale (100 points) Mean difference (95% CI)						GI Upset RR (95% CI)
							2.14 1.56 to 2.72						2.14 0.68 to 6.76
	Bettini 1986 Italy	Adult Outpatient Influenza‐like symptoms	Diclofenac 25 mg p.o. BD For 2 days (n = 60)	500 mg Aspirin p.o. TDS for 2 days (n = 60)	6 h Fever Mean Difference (95% CI)		2nd day sore throat moderate symptom reduction RR (95% CI)						GI Upset RR (95% CI)
					0.9 0.5 to 1.3		0.99 0.77 to 1.28						0.2 0.02 to 1.66
**Aspirin**	Voelker 2016 USA	Adult Outpatient URTI	1000 mg Aspirin p.o. single dose (n = 71)	Placebo (n = 36)			VAS^h^ Summed Pain Intensity Differences (0‐100mm, 0–2hr) mean difference (95% CI)						GI upset RR (95% CI)
							‐34.6 ‐46.77 to −22.43						
							First Pain Relief in minutes median (95% CI)						
							33.3 (28.9, 41.7)	90.8 (45.8, NA)					0.507 0.11 to 2.39
							Pain score Relieved by first dose Mean difference (95% CI)						
							RR= 1.73 1.26 to 2.38						

	Kor 2016 Mayo Clinic USA	Adult Inpatient ARDS	Aspirin 325mg I.V. day 1, followed by 81mg of I.V. daily up to day 7 (n = 195)	placebo (n = 195)					ARDS development within 7‐days RR (95% CI)		ICU length of stay Median (1st to 3rd quartile)		GI upset RR (95% CI)
											3.2 (1.8 to 6.0)	2.4 (1.6 to 5.2)	
									RR = 1.18 0.64 to 2.18		ICU length of stay Mean difference (95% CI)		
											0.20 ‐1.19 to 1.59		1 0.06 to 15.87
									Ventilator‐free to day 28 (ventilated patients) Median (Q1‐Q3)		Hospital length of stay Median (1st to 3rd quartile)		
											5.0 (3.0 to 10.0)	6.0 (4.0 to 10.0)	
									23.0 (17.0 to 26.0)	23.0 (9.0 to 26.0)	Hospital length of stay Mean difference (95% CI)		GI bleeding RR (95% CI)
											0.200 ‐1.81 to 2.21		
									Ventilator‐free to day 28 (ventilated patients) mean difference (95% CI)		Mortality 28‐days RR (95% CI)		
											1 0.49 to 2.04		1.5 0.25 to 8.88
									‐0.30 ‐1.13 to 1.73		Mortality 1‐year RR (95% CI)		
1.02 0.71 to 1.47													

Eccles 2013 U.K.	Adult Outpatient Sore throat	Aspirin 1000mg p.o. daily for 3 days (n = 239)	Placebo (n = 121)			Total pain relief (0‐3 score) 0‐4hr Mean difference (95% CI)						GI upset RR (95% CI)

−0.1 −0.11 to −0.09		1.82 0.69 to 4.79

	Eccles 2003 U.K./Sweden	Adult Outpatient URTI	Aspirin 800mg p.o. daily for 3 days (n = 139)	Placebo (n = 133)			Sum of pain intensity difference (0–3 score) 0–2hr Mean difference (95% CI)						GI upset RR (95% CI)
							−1.4 −2.01 to −0.79						1.59 0.39 to 6.54
	Broggini 1986 Italy	Adult Outpatient Influenza	Aspirin 500mg p.o. BD for 4 days (n = 15)	Flurbiprofen 100mg p.o. BD for 4 days (n = 15)	6 h fever temperature Mean difference (95% CI)	4th day Cough relief RR (95% CI)	4th day Pharyngeal pain relief RR (95% CI)						GI upset RR (95% CI)
					−0.10 −0.32 to 0.12	3.9 0.47 to 32.09	1.15 0.55 to 2.39						1.00 0.16 to 6.20
	Loose 2011 U.S.A.	Adult outpatient URTI	Aspirin 1000mg + 60mg Pseudoephedrine single dose p.o. (n = 161)	Placebo (n = 162)									GI upset RR (95% CI)
													1.00 0.25 to 3.95
	Bachert 2005 Russia/ Ukraine	Adult Outpatient URTI	Aspirin 1000mg p.o. single dose (n = 78)	Placebo (n = 78)	Feverish discomfort 0––6 h (0–10 score) Mean difference (95% CI)		Sore throat (0–10 score) Mean difference (95% CI)						GI upset RR (95% CI)
					−2.9 −3.00 to −2.80								
					Maximum temperature reduction in 6hr Mean difference (95% CI)		−0.49 −0.62 to −0.36						3.33 0.95 to 11.65
−1.04 −1.08 to −1.00													
	Barberi Italy 1993	Pediatric Inpatient/ Outpatient URTI/LRTI	Aspirin 360mg p.o. BD for 5 days (n = 35)	Nimesulide 50mg p.o. BD for 5 days (n = 35)		Day 2 Cough (0–3 score) Mean difference (95% CI)	Day 2 Pharyngeal hyperemia (0–3 score) Mean difference (95% CI)						GI upset RR (95% CI)
						−0.18 (−0.25 to −0.11)							
						Day 3 Cough (0–3 score) Mean difference (95% CI)	0 −0.14 to 0.14						
						−0.14 −0.22 to −0.06							
						Day 2 Dyspnea (0–3 score) Mean difference (95% CI)	Day 3 Pharyngeal hyperemia (0–3 score) Mean difference (95% CI)						2.67 0.77 to 9.23
						−0.22 −0.37 to −0.07							
						Day 3 Dyspnea (0–3 score) Mean difference (95% CI)	−0.08 −0.21 to 0.05						
						−0.34 −0.49 to −0.19							
	Kondoth 1984 India	Pediatric Inpatient/ Outpatient Fever	Aspirin 15 mg/kg daily for 2 days (n = 14)	Ibuprofen 7mg/kg daily for 2 days (n = 14)	6 h fever reduction (˚C) in patients with URTI Mean difference (95% CI)								Total AE RR (95% CI)
					−0.33 −0.13 to −0.53								
					4 h fever reduction (˚C) in patients with URTI Mean difference (95% CI)								1 0.02 to 47.19
					−0.57 −0.42 to −0.72								
**Other NSAIDs**	De Looze, F. 2018 Australia	Adult Outpatient URTI	Flurbiprofen 8.75 mg spray (three puffs), not allowed to redose for 6 hr, then redose every 3–6 h up to 5 doses/day for 3 days, as required (n = 249)	Placebo spray (n = 256)			Number of patients with at least moderate relief at 75mins RR (95% CI)						
							1.96 1.45 to 2.64						
							6 h TOTPAR sore throat pain relief						
							−0.77 −0.80 to −0.74						
	Azuma, A. 2010 Japan	Adult Outpatient URTI	Zaltoprofen 160 mg p.o. single dose (n = 56)	Placebo (n = 57)	6 h body temperature Mean difference (95% CI)		Visual Analogue Scale (0–100 score) sore throat first dose Mean difference (95% CI)						
					−0.4 −0.45 to −0.35		−7.80 −9.20 to −6.40						
	Goto, M. 2007 Japan	Adult Outpatient URTI	Loxoprofen sodium 60 mg p.o. BD for 7 days (n = 84)	Placebo (n = 90)	Fever duration days Mean difference (95% CI)	Cough duration days Mean difference (95% CI)	Sore throat Duration (days) Mean difference (95% CI)				Illness duration days Mean difference (95% CI)		Number of patients with all adverse events (not confined to GI) RR (95% CI)
					0.04 −0.71 to 0.79								
					maximum body temperature Mean difference (95% CI)	−0.62 −1.84 to 0.60	−0.46 −1.42 to 0.50				−0.55 −1.54 to 0.44		8.57 1.10 to 67.09
					0 −0.23 to 0.23								
	Weckx, L. 2002 Latin America	Adult Outpatient Pharyngitis	Celecoxib 200mg p.o. BD for 5 days (n = 117)	Diclofenac 75mg p.o. BD for 5 days (n = 101)									GI upset RR (95% CI)
													0.3837 0.1218 to 1.2085

	Passali, D 2001 Italy	Adult Outpatient Pharyngitis	Mouthwash Ketoprofen lysine salt 160mg gargled twice daily (until pain remission or up to 7 days) (n = 119)	Mouthwash Benzydamine hydrochloride 22.5mg gargled twice daily (until pain remission or up to 7 days) (n = 120)			No. of patients with Pain reduced to mild/ absent RR (95% CI)						Dry mouth RR (95% CI)
							1.04 0.98 to 1.09						
							No. of patients who completely recovered RR (95% CI)						0.46 0.27 to 0.79
							1.12 0.95 to 1.32						
	Ebel 1985 U.S.A.	Adult Inpatient URTI	Sulindac 200mg BD for 7 days (n = 156)	Placebo (n = 156)	No. with >1˚C fever reduction day 1 RR (95% CI)								GI upset RR (95% CI)
					1.68 1.21 to 2.35								
					No. with >1˚C fever reduction day 2 RR (95% CI)								
					1.21 0.98 to 1.48								2 1.10 to 3.65
					Day7 no. without fever RR (95% CI)								
1.26 1.11 to 1.43													

	Choi, S.J. 2018 South Korea	Pediatric Inpatient URTI	Propacetamol IV/inf. <10 kg : 15 mg/kg >10 kg : 30 mg/kg Single dose (n = 125)	Dexibuprofen 6 mg/kg IV/inf. Stat + Placebo (n = 138)	6 h No. of Normalized Fever RR (95% CI)								GI upset RR (95% CI)
					1.25 0.75 to 2.07								
					4 h Fever Mean difference (95% CI)								
					0.19 −0.03 to 0.41								0.46 0.17 to 1.27
					6 h Fever Mean difference (95% CI)								
0.01 −0.16 to 0.18													

Interventions: BD, two doses daily; TDS, three doses per day; QID, four doses per day; p.o., oral intake; I.V., intra venous; Inf., infusion; Stat., immediately.

Abbreviations: ARDS: Acute Respiratory Distress Syndrome; ARTI, acute respiratory tract infection; CI, Confidence Interval; GI, Gastrointestinal; ICU, intensive care unitRR, Relative Risk; RSV, Respiratory Syncytial Virus; URTI, upper respiratory tract infection; VAS, visual analogue scale.

Ibuprofen was investigated in 12 studies: 7 studies included adults with acute viral respiratory infections and 5 assessed children with viral ARTI; all of these studies were conducted in the outpatient setting. Among the 4 studies that assessed naproxen, 1 study included children and 3 studies included adults. All studies in the diclofenac group included adults in the outpatient setting only. In the aspirin group, 7 studies included adult patients with viral ARTI, of which 6 were conducted in an outpatient setting and 1 in an inpatient setting. Two studies included children with viral ARTI without specifying the setting.

Sulindac, celecoxib, loxoprofen, ketoprofen, dexibuprofen, flurbiprofen, and zaltoprofen were grouped together as ‘Other NSAIDs’ because each medicine had only 1 RCT. Two studies in this group included inpatient participants: 1 included adults and 1 included children. The 5 remaining studies evaluated adults in outpatient settings.

### 3.2 Risk of bias

Appendices 2–6 present the risk of bias of individual studies for each of the NSAIDs. The overall risk of bias for ibuprofen and diclofenac studies was low. In the naproxen group, the risk of bias was unclear for 3 studies and low for 1 study.^13^ In aspirin studies, only 2 out of 9 studies provided details about blinding of outcome assessment, therefore, the risk of bias due to inadequate blinding of outcome assessment is unclear. The overall risk of bias in the aspirin studies was unclear to low (Appendices 2–6).

### 3.3 Findings

Below, we present an overview of the effects of each of the NSAIDs on our outcomes: fever, cough, sore throat, respiratory distress, hospital stay, and adverse events.

#### 3.3.1 Ibuprofen

Twelve studies that assessed the efficacy of Ibuprofen on signs and symptoms of ARTI were finally included in our systematic review. Tables 2 and 3 present the results of each of these studies.^14^, ^15^, ^16^, ^17^, ^18^, ^19^, ^20^, ^21^, ^22^, ^23^, ^24^, ^25^

**TABLE 2:** Summary of findings: Ibuprofen compared to Placebo/Other NSAIDs for adult ARTI

**Patient or population**: adult ARTI **Intervention**: Ibuprofen **Comparison**: Placebo/Other NSAIDs						
Outcome № of participants (studies)	Relative effect (95% CI)	Anticipated absolute effects (95% CI)			Certainty	What happens
				Difference		
Fever № of participants: 535 (2 RCTs)	–	not pooled	–	not pooled	⨁⨁⨁⨁ HIGH	Ibuprofen results in a moderate reduction in fever.
Cough № of participants: 450 (3 RCTs)	–	not pooled	–	not pooled	⨁⨁◯◯ LOW ^a,b,c^	The evidence suggests that ibuprofen results in little to no difference in cough. Use of different, non–uniform variables for cough hinders accumulative reasoning. The little effect shown by Gwaltney et al. may be due to the additional Chlorpheniramine. Winther et al. study results show a paradoxical increase of symptom severity in patients who initially had a mild cough.
Sore Throat № of participants: 355 (4 RCTs)	–	not pooled	–	not pooled	⨁⨁◯◯ LOW ^d,e,f^	Ibuprofen likely results in a reduction in Sore Throat pain. The effect seems to be larger if the pain is more prominent upon presentation.
ARDS (minute‐ventilation) follow up: mean 30 days № of participants: 455 (1 RCT)	–	The mean ARDS (minute‐ventilation) was **14.13** lit/min	–	mean **0.92 lit/min lower** (0.8453 lower to 0.9947 lower)	⨁⨁◯◯ LOW ^g^	Ibuprofen may result in a slight reduction in Oxygen therapy needed for ICU admitted patients with sepsis. it is worthy of note that the initially required oxygen volume is approximately 13 liters in both study arms.
Length of Stay ‐ not reported	–	–	–	–	–	No study investigated the efficacy of Ibuprofen in the disease duration of the adult population.
Mortality follow up: mean 30 days № of participants: 455 (1 RCT)	**RR 0.88** (0.69 to 1.10)	40.3%	**35.4%** (27.8 to 44.3)	**4.8% fewer** (12.5 fewer to 4 more)	⨁⨁◯◯ LOW ^h^	Ibuprofen may result in little to no difference in mortality.
Gastrointestinal Adverse Events (GI upset) № of participants: 1467 (5 RCTs)	not pooled	19.9%	not pooled	not pooled	⨁⨁◯◯ LOW ^i,j^	Even though the total incidence of adverse events is large, there is considerable variation in the imprecision of results across studies. Overall, the evidence suggests little to no difference in gastrointestinal adverse events in adults in short terms.

*GRADE Working Group grades of evidence*

**High certainty:** We are very confident that the true effect lies close to that of the estimate of the effect

**Moderate certainty:** We are moderately confident in the effect estimate: The true effect is likely to be close to the estimate of the effect, but there is a possibility that it is substantially different

**Low certainty:** Our confidence in the effect estimate is limited: The true effect may be substantially different from the estimate of the effect

**Very low certainty:** We have very little confidence in the effect estimate: The true effect is likely to be substantially different from the estimate of effect

Explanations: a. Winther et al. study resulted in an increase in cough severity score, whereas Gwaltney et al. reported a minor decrease in symptom severity. Llor et al. reported a decrease in symptom duration; b. Gwaltney et al. used Chlorpheniramine along with Ibuprofen; c. Llor et al. reported symptom duration rather than symptom severity d. Bouroubi et al. demonstrated a moderate alleviating effect, whereas Gwaltney et al. showed a small effect size. Meanwhile, Sperber et al. concluded a mixed effect that may be due to its relatively smaller sample size. Winther et al. reported a mildly worsening effect probably due to initial selection bias; e. Gwaltney et al. uses concomitant chlorpheniramine. Sperber et al. uses concomitant pseudoephedrine; f. One study by Sperber et al. resulted in a variable effect due to the small sample size; g. Bernard et al. measured minute‐ventilation in sepsis patients of varied etiology including but not confined to pneumonia; h. Bernard et al. recorded 30‐day mortality in sepsis patients of varied etiology including but not confined to pneumonia; i. In Adults: Sperber et al., and Bernard et al report a protective RR, whereas Little et al., Llor et al., and Bouroubi et al. report a harmful RR and j. Most calculated 95% confidence intervals for RRs are extremely wide. Sperber et al. had a small sample size.

Abbreviations: CI, Confidence interval; RR, Risk ratio.

* **The risk in the intervention group** (and its 95% confidence interval) is based on the assumed risk in the comparison group and the **relative effect** of the intervention (and its 95% CI).

**TABLE 3:** Summary of findings: Ibuprofen compared to Placebo/Other NSAIDs for Pediatric ARTI

**Patient or population**: Pediatric ARTI **Intervention**: Ibuprofen **Comparison**: Placebo/Other NSAIDs						
Outcome № of participants (studies)	Relative effect (95% CI)	Anticipated absolute effects (95% CI)			Certainty	What happens
				Difference		
Fever № of participants: 483 (4 RCTs)	–	not pooled	–	not pooled	⨁⨁⨁⨁ HIGH	Ibuprofen results in a large reduction in fever.
Cough follow up: 2 days № of participants: 52 (1 RCT)	**RR 0.79** (0.60 to 1.06)	89.3%	**70.5%** (53.6 to 94.6)	**18.7% fewer** (35.7 fewer to 5.4 more)	⨁⨁◯◯ LOW ^a,b^	Ibuprofen likely results in little to no difference in cough. small effect is seen here may be due to additional Anti‐biotics.
Sore Throat follow up: 2 days № of participants: 153 (1 RCT)	**RR 1.68** (1.27 to 2.23)	44.7%	**75.2%** (56.8 to 99.8)	**30.4% more** (12.1 more to 55 more)	⨁⨁⨁◯ MODERATE ^b^	Although Penicillin is used in both groups, the Ibuprofen group had a significant reduction in sore throat. Ibuprofen likely results in a large decrease in pediatric sore Throat.
ARDS ‐ not reported	–	–	–	–	–	No studies investigated the efficacy of viral ARDS in the pediatric population.
Length of Stay follow up: 5 days № of participants: 104 (1 RCT)	**RR 0.83** (0.49 to 1.38)	40.4%	**33.5%** (19.8 to 55.7)	**6.9% fewer** (20.6 fewer to 15.3 more)	⨁⨁⨁◯ MODERATE ^c^	No significant difference in the recovery of the pediatric common cold was observed between Ibuprofen and Paracetamol.
Gastrointestinal adverse events (GI AE) № of participants: 579 (4 RCTs)	not pooled	0.0%	not pooled	not pooled	⨁⨁⨁◯ MODERATE ^d^	The overall incidence of gastrointestinal discomfort is large in children. In short periods of use, Ibuprofen does not significantly cause higher gastrointestinal adverse events than Dexibuprofen or Paracetamol.

*GRADE Working Group grades of evidence*:

**High certainty:** We are very confident that the true effect lies close to that of the estimate of the effect

**Moderate certainty:** We are moderately confident in the effect estimate: The true effect is likely to be close to the estimate of the effect, but there is a possibility that it is substantially different

**Low certainty:** Our confidence in the effect estimate is limited: The true effect may be substantially different from the estimate of the effect

**Very low certainty:** We have very little confidence in the effect estimate: The true effect is likely to be substantially different from the estimate of effect

Explanations: a. no blinding of participants and personnel; b. Additional use of Anti‐Biotics; c. small sample size and d. All 4 studies are against other interventions rather than placebo.

Abbreviations: CI, Confidence interval; RR, Risk ratio.

* **The risk in the intervention group** (and its 95% confidence interval) is based on the assumed risk in the comparison group and the **relative effect** of the intervention (and its 95% CI).

##### Fever

Four studies investigated the effectiveness of ibuprofen for fever control in children in the outpatient setting. Kim et al^19^ compared ibuprofen with dexibuprofen and found that ibuprofen reduced fever by 1.38˚C ± 0.84˚C and was not significantly different from dexibuprofen. Yoon et al^25^ compared ibuprofen with dexibuprofen for fever reduction during a 6‐hour period and found that ibuprofen reduced fever by 0.9˚C ± 0.9˚C and was not significantly different from dexibuprofen. Hay et al^18^ measured the onset of action for ibuprofen compared with paracetamol and found that ibuprofen started acting 28 min faster than paracetamol (MD, 28.8; 95% CI, 7.68‐49.92). Ulukol et al^23^ found no statistically significant difference between ibuprofen and paracetamol for fever control (RR, 0.6; 95% CI, 0.33–1.12).

Two studies assessed the effects of ibuprofen on fever in adults with viral respiratory tract infections. Bernard et al^14^ demonstrated a mean 0.8˚C (95% CI, 0.78‐0.82) reduction in patients who were hospitalized with sepsis. Winther and Mygind^24^ reported a 0.6 (95% CI, 0.51–0.75) points reduction of chilliness severity (on a scale of 0–3) and a significant temperature reduction in adults in the outpatient setting.

##### Cough

Three studies investigated the efficacy of ibuprofen in adults with coughs. Llor et al^21^ recorded the number of days each patient continued to cough in hospitalized patients. The intervention group had coughs for about 9 (95% CI,7.5–10.5) days, which was about 2 (95% CI,1.5 to 2.5) days shorter than the placebo group. Winther and Mygind^24^ reported a nonsignificant improvement of cough severity score in a 3‐day follow‐up of outpatients compared with placebo (MD, 0.83; 95% CI, 0.61–1.05). Gwaltney^17^ reported a relative increase in coughs with ibuprofen prescription in patients who were inoculated with Rhinovirus (MD, 0.12; 95% CI, 0.09‐0.15).

Ulokol et al^23^ compared the effects of paracetamol and ibuprofen in reducing coughs in children. They found that while children in both groups experienced an improvement in coughs, a slightly higher number of participants (albeit nonsignificant) in the paracetamol group experienced cough improvement (RR, 0.79; 95% CI, 0.60–1.06).

##### Sore throat

Four studies investigated ibuprofen effects on the sore throat in adults. Two out of 4 studies used ibuprofen as an add‐on to other drugs. Gwaltney^17^ used concomitant chlorpheniramine in the intervention group; Sperber et al^22^ in 1989 used pseudoephedrine in addition to ibuprofen for both groups. Both of these studies showed a significant reduction in sore throat severity. On the contrary, Winther and Mygind^24^ did not find a significant effect.

Bertin et al^15^ reported a significant pain relief when ibuprofen was added to penicillin for sore throat in children (RR, 1.68; 95% CI, 1.27–2.23). Overall, 4 of 5 studies reported that ibuprofen effectively reduced sore throat.

##### ARDS

Only one study investigated ibuprofen use in ARDS. Bernard et al^14^ used Ibuprofen in 455 intensive care units (ICU)‐admitted adults with sepsis. Ibuprofen decreased the need for oxygenation by 0.92 liters/min (95% CI, 0.85–0.99) to a mean of 13 L/min.

##### Length of stay

Hay et al^18^ investigated the clinical recovery of outpatient children from the disease on the second and fifth day; 33% and 40% of patients in ibuprofen and paracetamol groups recovered, respectively. We did not find any study to investigate LOS and ibuprofen in adults.

##### Mortality

Only 1 study assessed mortality as an outcome with the use of ibuprofen. Bernard et al^14^ reported a nonsignificant small reduction in mortality.

##### Gastrointestinal adverse event

Five studies in the adult population reported gastrointestinal (GI) adverse events with ibuprofen in ARTI. Bernard et al^14^ recorded GI bleeding in patients with sepsis, suggesting a small nonsignificant protective effect (RR, 0.58; 95% CI, 0.26 to 1.28). Little et al^20^ compared ibuprofen with paracetamol in 302 patients. The overall number of adverse events for ibuprofen was 75 in 150 patients compared with 94 in 152 patients for paracetamol. Llor et al^21^ and Bouroubi et al^16^ showed a nonsignificant increased risk of gastrointestinal adverse events with ibuprofen. Overall, effect estimates for this outcome were very imprecise.

Four studies assessed the effects of ibuprofen on rates of gastrointestinal adverse events in children with ARTI. These studies had different intervention durations, ranging from a single dose in 2 studies to 7 days in another. These studies compared the effects of either dexibuprofen, paracetamol, or pseudoephedrine with ibuprofen in children. Kim et al^19^ reported that 35 patients out of 75 in the ibuprofen group experienced GI upset (nausea, vomit, diarrhea, etc.). In the protective immunity from T cells to covid‐19 in health workers (PITCH) study by Hay et al.,^18^ a total of 12 in 52 participants had GI problems. Yoon et al^25^ and Bertin et al^15^ reported fewer incidents—7 in 85 and 5 in 77, respectively.

#### 3.3.2 Naproxen

Four studies investigated the efficacy of naproxen in ARTIs, and the results are presented in Table 4.^13^, ^26^, ^27^, ^28^

**TABLE 4:** Summary of findings: Naproxen compared to Placebo/Other NSAIDs for ARTI

**Patient or population**: ARTI **Intervention**: Naproxen **Comparison**: Placebo/Other NSAIDs						
Outcome № of participants (studies)	Relative effect (95% CI)	Anticipated absolute effects (95% CI)			Certainty	What happens
				Difference		
Fever № of participants: 79 (1 RCT)	**RR 0.1368** (0.0315 to 0.5936)	4.1%	**0.6%** (0.1 to 2.5)	**3.6% fewer** (4 fewer to 1.7 fewer)	⨁⨁⨁⨁ HIGH ^a^	Naproxen results in a reduction in fever.
Cough № of participants: 79 (1 RCT)	–	The mean cough was **0**	–	MD **0.8 lower** (1.8 lower to 0.17 higher)	⨁⨁⨁◯ MODERATE ^b^	Naproxen results in little to no difference in cough compared with placebo.
Sore Throat № of participants: 79 (1 RCT)	–	The mean sore Throat was **0**	–	MD **0.5 lower** (1.9 lower to 0.93 higher)	⨁⨁⨁◯ MODERATE ^b^	Naproxen probably results in little to no difference in sore Throat.
ARDS № of participants: 217 (1 RCT)	**RR 0.5266** (0.3345 to 0.8288)	37.3%	**19.6%** (12.5 to 30.9)	**17.6% fewer** (24.8 fewer to 6.4 fewer)	⨁⨁◯◯ LOW ^c^	Naproxen may result in a reduction in ARDS.
Mortality № of participants: 217 (1 RCT)	**RR 0.1142** (0.0147 to 0.8862)	8.2%	**0.9%** (0.1 to 7.3)	**7.2% fewer** (8.1 fewer to 0.9 fewer)	⨁⨁⨁◯ MODERATE ^d^	Combination of Naproxen + Clarithromycin may result in a reduction in mortality. 1/107 compared with 9/110 were deceased in a trial of Naproxen + Clarithromycin + Oseltamivir versus Oseltamivir alone in H3N2 influenza patients followed for 30 days.
GI Adverse Events № of participants: 103 (2 RCTs)	not pooled	10.4%	not pooled	not pooled	⨁⨁◯◯ LOW ^e^	The effect of Naproxen in GI upset is uncertain. Salmon Rodriguez et al. in a pediatrics population‐although imprecise‐ reports inferior tolerability compared with Nimesulide.

*GRADE Working Group grades of evidence*:

**High certainty:** We are very confident that the true effect lies close to that of the estimate of the effect

**Moderate certainty:** We are moderately confident in the effect estimate: The true effect is likely to be close to the estimate of the effect, but there is a possibility that it is substantially different

**Low certainty:** Our confidence in the effect estimate is limited: The true effect may be substantially different from the estimate of the effect

**Very low certainty:** We have very little confidence in the effect estimate: The true effect is likely to be substantially different from the estimate of effect

Explanations: a. fever in this study is not reported as mean temperature. A 4‐score subjective scale and consecutive dichotomous temperature measurement are reported; b. The total number of participants is below 100; c. Surrogate outcomes are used for ARDS. a co‐intervention of clarithromycin was used along with naproxen; d. A co‐intervention of clarithromycin was used along with naproxen and e. Effect estimates in 3 studies demonstrate a wide confidence interval; this could be due to a sample size of less than 100 patients per trial. Also, effect estimates are not consistent in larger trials.

Abbreviations: CI, Confidence interval; MD, Mean difference; RR, Risk ratio.

* **The risk in the intervention group** (and its 95% confidence interval) is based on the assumed risk in the comparison group and the **relative effect** of the intervention (and its 95% CI).

##### Fever

Sperber et al^28^ examined the effect of naproxen on subjective chilliness score (0–4) and the presence of fever on individuals inoculated with rhinovirus. They reported that naproxen effectively prevented fever (RR, 0.14; 95% CI, 0.03 to 0.59) and improved chilliness scores in a 4‐day trial.

##### Cough

Sperber et al^28^ reported a nonsignificant improvement in cough with naproxen in adult patients inoculated with rhinovirus (MD:0.8 95% CI, −0.17 to 1.80).

##### Sore throat

Sore throat was investigated in three studies. However, because of inadequate reporting, we were unable to determine effect estimates. In the Sperber et al trial,^28^ naproxen did not significantly relieve sore throat (MD, 0.5; 95% CI, −0.93 to 1.9).

##### ARDS

Hung et al^13^ used a combination of naproxen, clarithromycin, and oseltamivir in patients with influenza compared with oseltamivir alone. Patients who received the drug combination were less likely to require ventilator support (RR, 0.53; 95%CI, 0.33–0.83).

##### Length of stay

Patients who received clarithromycin and naproxen as an add‐on to oseltamivir in the Hung et al trial^13^ were less likely to need intensive treatment at the hospital (RR, 0.51; 95% CI, 0.31–0.86) and intensive care unit (ICU) admissions (RR, 0.29; 95% CI, 0.06 to 1.38).

##### Mortality

Hung et al^13^ reported a reduction in 30‐day and 90‐day mortality ((RR, 0.11; 95% CI, 0.01–0.8) for 30‐day mortality) in the group who received naproxen, clarithromycin, and oseltamivir compared with oseltamivir alone.

##### Gastrointestinal adverse events

Two studies reported gastrointestinal outcomes in outpatient adults, but their results were extremely imprecise because they had very small sample sizes. Rodriguez et al^27^ compared the tolerability of naproxen with nimesulide in children; their results showed a higher incidence of gastrointestinal adverse events in the naproxen group (RR, 2.73; 95% CI, 0.95–7.89).

#### 3.3.3 Diclofenac

Three studies investigated the efficacy of diclofenac in ARTIs.^29^, ^30^, ^31^

##### Fever

Two studies investigated fever control using diclofenac. In the study by Bettini et al,^29^ diclofenac reduced fever from 38.7 (0.4) to 37.4 (0.5). The antipyretic effects lasted 2 h longer than aspirin. Grebe et al^30^ reported that a significantly greater proportion of patients experienced fever relief after 6 h (RR, 2.73; 95% CI, 1.27–5.86) with diclofenac compared with placebo in Table 5.

**TABLE 5:** Summary of findings: Diclofenac compared to Placebo/Other NSAIDs for ARTI

**Patient or population**: ARTI **Intervention**: Diclofenac **Comparison**: Placebo/Other NSAIDs						
Outcome № of participants (studies)	Relative effect (95% CI)	Anticipated absolute effects (95% CI)			Certainty	What happens
				Difference		
Fever № of participants: 356 (2 RCTs)	–	not pooled	–	not pooled	⨁⨁⨁⨁ HIGH	Diclofenac reduces fever.
Cough ‐ not reported	–	–	–	–	–	No studies were found.
Sore Throat № of participants: 328 (2 RCTs)	–	not pooled	–	not pooled	⨁⨁⨁⨁ HIGH	Diclofenac results in a large reduction in sore throat. The effect size is similar to celecoxib.
ARDS ‐ not reported	–	–	–	–	–	No studies were found.
Length of stay/ Mortality ‐ not reported	–	–	–	–	–	No studies were found.
GI Upset № of participants: 596 (3 RCTs)	not pooled	3.8%	not pooled	not pooled	⨁⨁◯◯ LOW ^a,b^	Diclofenac likely does not increase GI upset.

*GRADE Working Group grades of evidence*:

**High certainty:** We are very confident that the true effect lies close to that of the estimate of the effect

**Moderate certainty:** We are moderately confident in the effect estimate: The true effect is likely to be close to the estimate of the effect, but there is a possibility that it is substantially different

**Low certainty:** Our confidence in the effect estimate is limited: The true effect may be substantially different from the estimate of the effect

**Very low certainty:** We have very little confidence in the effect estimate: The true effect is likely to be substantially different from the estimate of effect

Explanations: a. The effect estimates are large and considerably vary across studies and b. Results from all three studies are wide in confidence intervals including large effects in both directions.

Abbreviations: CI, Confidence interval.

* **The risk in the intervention group** (and its 95% confidence interval) is based on the assumed risk in the comparison group and the **relative effect** of the intervention (and its 95% CI).

Overall, diclofenac effectively reduced fever in adults with ARTI symptoms.

##### Cough

No studies in the diclofenac group investigated cough‐related outcomes.

##### Sore throat

Two studies investigated the effectiveness of diclofenac in the treatment of sore throat. Bettini et al^29^ compared the efficacy of diclofenac with aspirin; 21 of 23 patients with sore throat reported improvement of their throat pain. Weckx et al^31^ reported that diclofenac and celecoxib both significantly reduced sore throat in 208 adults with influenza‐like symptoms.

##### ARDS, LOS, and mortality

No studies involving diclofenac investigated these outcomes.

##### Gastrointestinal adverse events

Three studies investigated GI outcomes. Weckx et al^31^ reported a nonsignificant higher rate of GI adverse events in the diclofenac group (9/123) compared with celecoxib (4/117). Grebe et al^30^ reported a lower risk for diclofenac compared with placebo. However, the event rates in this study were limited to 1 to 2 cases per arm. Bettini et al^29^ observed 5 events in the aspirin group compared with 1 event in the diclofenac group.

#### 3.3.4 Aspirin

Nine studies investigated the efficacy of aspirin in ARTIs and the results are presented in Tables 6 and 7.^32^, ^33^, ^34^, ^35^, ^36^, ^37^, ^38^, ^39^, ^40^

**TABLE 6:** Summary of findings: Aspirin compared to Placebo/Other NSAIDs for adult ARTI

**Patient or population**: adult ARTI **Intervention**: Aspirin **Comparison**: Placebo/Other NSAIDs						
Outcome № of participants (studies)	Relative effect (95% CI)	Anticipated absolute effects (95% CI)			Certainty	What happens
				Difference		
Fever follow up: range single dose to 4 days № of participants: 186 (2 RCTs)	–	not pooled	–	not pooled	⨁⨁⨁◯ MODERATE ^a^	Aspirin results in a large reduction in fever.
Cough follow up: 4 days № of participants: 23 (1 RCT)	**RR 3.90** (0.47 to 32.09)	7.7%	**30.0%** (3.6 to 100)	**22.3% more** (4.1 fewer to 239.2 more)	⨁◯◯◯ VERY LOW ^b,c^	Aspirin may have little to no effect on cough but the evidence is very uncertain.
Sore Throat follow up: range 6 hours to 4 days № of participants: 905 (5 RCTs)	‐	not pooled	‐	not pooled	⨁⨁⨁◯ MODERATE ^d,e^	Aspirin likely results in a slight reduction in sore throat pain.
ARDS № of participants: 390 (1 RCT)	**RR 1.1765** (0.6358 to 2.1768)	8.7%	**10.3%** (5.5 to 19)	**1.5% more** (3.2 fewer to 10.3 more)	⨁⨁⨁◯ MODERATE ^f^	Aspirin did not decrease ARDS in a single study by Kor et al.
Length Of Stay № of participants: 390 (1 RCT)	–	The mean length Of Stay was **0** days	–	mean **0.2 days fewer** (1.8114 fewer to 2.2114 more)	⨁⨁⨁◯ MODERATE ^g^	Aspirin resulted in little to no difference in Hospital length Of Stay in a single study by Kor et al.
Mortality № of participants: 390 (1 RCT)	**RR 1.00** (0.49 to 2.04)	7.2%	**7.2%** (3.5 to 14.6)	**0.0% fewer** (3.7 fewer to 7.5 more)	⨁⨁⨁◯ MODERATE ^h^	Aspirin results in little to no difference in mortality.
Gastrointestinal Adverse Events follow up: range 1 dose to 4 days № of participants: 1248 (6 RCTs)	**RR 1.54** (0.92 to 2.60)	3.7%	**5.7%** (3.4 to 9.5)	**2.0% more** (0.3 fewer to 5.9 more)	⨁⨁◯◯ LOW ^i^	Gastrointestinal adverse events were not found to be statistically different between groups, but the Aspirin group tended to experience more events

*GRADE Working Group grades of evidence*:

**High certainty:** We are very confident that the true effect lies close to that of the estimate of the effect

**Moderate certainty:** We are moderately confident in the effect estimate: The true effect is likely to be close to the estimate of the effect, but there is a possibility that it is substantially different

**Low certainty:** Our confidence in the effect estimate is limited: The true effect may be substantially different from the estimate of the effect

**Very low certainty:** We have very little confidence in the effect estimate: The true effect is likely to be substantially different from the estimate of effect

Explanations: a. Allocation concealment of studies poses a high risk of bias; outcome assessment was unclear in both studies; b. methods of randomization and blinding are not explained. study protocols are not available to assess the completeness of outcome reporting; c. A Small number of participants; d. 2 out of 5 studies lack explanation on randomization and concealment protocols. On the contrary, the remaining 3, make up the majority of the sample population and are well conducted; e. Although Voelker et al. and Eccles 2013 et al. report a moderate effect on a sore throat, the rest of the studies report little to no effect; f. the Confidence Interval on the relative risk suggests both a moderate reduction and an increase in the incidence of ARDS; g. the effect estimate includes a wide range of effects, both increase and reduce in LOS; h. ARDS patients are not confined to viral pneumonia and i. Both large positive and negative effects with wide confidence intervals are reported.

Abbreviations: CI, Confidence interval; RR, Risk ratio.

* **The risk in the intervention group** (and its 95% confidence interval) is based on the assumed risk in the comparison group and the **relative effect** of the intervention (and its 95% CI).

**TABLE 7:** Summary of findings: Aspirin compared to Placebo/Other NSAIDs for Pediatric ARTI

**Patient or population**: Pediatric ARTI **Intervention**: Aspirin **Comparison**: Placebo/Other NSAIDs						
Outcome № of participants (studies)	Relative effect (95% CI)	Anticipated absolute effects (95% CI)			Certainty	What happens
				Difference		
Fever Reduction follow up: 6 hours № of participants: 28 (1 RCT)	–	The mean fever Reduction was **0.86 (0.21)** degree	–	MD **0.33 degree lower** (0.13 lower to 0.55 lower)	⨁⨁⨁◯ MODERATE ^a^	Aspirin probably results in a large reduction in fever.
Cough follow up: 3 days № of participants: 70 (1 RCT)	–	The mean cough was **2.00 (0.15)** (0–3 severity score)	–	MD **0.14 (0‐3 severity score) lower** (0.22 lower to 0.06 higher)	⨁⨁⨁◯ MODERATE ^b^	Aspirin does not reduce cough.
Sore Throat ‐ not reported	–	–	–	–	–	No studies investigated efficacy of Aspirin in pediatric sore throat pain relief.
ARDS ‐ not reported	–	–	–	–	–	No studies investigated efficacy of Aspirin in pediatric ARDS related outcomes.
Length of Stay ‐ not reported	–	–	–	–	–	No studies investigated efficacy of Aspirin in pediatric hospitalization and length of stay.
Gastrointestinal adverse events (GI AE) follow up: 7 days № of participants: 98 (2 RCTs)	not pooled	0.0%	not pooled	not pooled	⨁◯◯◯ VERY LOW ^a,b,c^	The evidence is very uncertain about the effect of aspirin on gastrointestinal adverse events.

*GRADE Working Group grades of evidence*:

**High certainty:** We are very confident that the true effect lies close to that of the estimate of the effect

**Moderate certainty:** We are moderately confident in the effect estimate: The true effect is likely to be close to the estimate of the effect, but there is a possibility that it is substantially different

**Low certainty:** Our confidence in the effect estimate is limited: The true effect may be substantially different from the estimate of the effect

**Very low certainty:** We have very little confidence in the effect estimate: The true effect is likely to be substantially different from the estimate of effect

Explanations: a. lacking information on allocation and randomization, also the intervention was not blinded to participants nor the examiners;

b. blinding, randomization and concealment not explained and c. very small sample sizes.

Abbreviations: CI, Confidence interval; MD, Mean difference.

* **The risk in the intervention group** (and its 95% confidence interval) is based on the assumed risk in the comparison group and the **relative effect** of the intervention (and its 95% CI).

##### Fever

Three articles assessed fever reduction for aspirin and all showed a significant reduction of fever.^32^, ^34^, ^37^ Broggini et al^34^ reported a large effect in fever reduction in adults similar to flurbiprofen (MD, 0.1˚C; 95% CI, −0.32 to 0.12). Also, Bachert et al^32^ reported that high‐dose aspirin reduced fever by 1.67˚C (95% CI, 1.53‐1.80). In the study by Kandoth et al,^37^ although both aspirin and ibuprofen reduced fever by about 1˚C in children, the effects were more prominent for ibuprofen compared with aspirin (MD, 0.57˚C; 95% CI, 0.42–0.72).

##### Cough

Barberi et al^33^ reported that day 2 and day 3 cough severity scores (on a scale of 0–3) reduced from 2.03 ± 0.15 to 1.30 ± 0.11 in the aspirin group. Broggini et al^34^ reported a moderate cough relief for aspirin in 24 adult patients with influenza compared to flurbiprofen.

##### Sore throat

Six studies assessed the effects of aspirin on sore throat using a variety of measures and tools.^32^, ^36^, ^40^ Voelker et al^40^ and Eccles et al^35^ reported a moderate reduction in pain, whereas the rest of the studies did not show a significant difference with placebo.

##### ARDS, mortality, and LOS

The study by Kor et al in 2016,^38^ assessed the effects of aspirin compared with placebo in 390 adult patients at risk of ARDS. The study did not show a significant reduction in respiratory distress, ICU admission, ventilation support, or mortality with aspirin.

##### GI adverse events

Seven studies investigated GI adverse events in adults.^32^, ^34^, ^35^, ^36^, ^38^, ^39^, ^40^ Kor et al^38^ reported that gastrointestinal bleeding was not significantly related to a 7‐day intravenous use of low‐dose aspirin. These 7 studies showed differing results with great imprecision.

We pooled six studies for the meta‐analysis that used 1000 mg aspirin for up to 3 days on adult patients in the outpatient setting. No significant heterogeneity was observed among the studies (*I^2^* = 0%). The *P*‐value for the chi‐square test was 0.53, and no asymmetry was observed in the funnel plot. Aspirin did not show significant increase in gastrointestinal adverse events (RR, 1.54; 95% CI, 0.92–2.60) (Appendices No. 7 and 8).

In children, two studies reported GI adverse events. Barberi et al^33^ reported a higher rate of GI adverse events (RR, 2.67; 95% CI, 0.77–9.23); and Kandoth^37^ reported no events in either group. Both studies had very small sample sizes.

### 3.4 Other NSAIDs

Table 1 demonstrates the summarized results of other NSAIDs.^31^, ^41^, ^42^, ^43^, ^44^, ^45^, ^46^, Two studies included inpatient participants: one in adults and one in children. Five remaining studies were conducted in adult outpatient settings. Sulindac, celecoxib, loxoprofen, ketoprofen, dexibuprofen, flurbiprofen, and zaltoprofen were studied.

## 4 DISCUSSION

The findings of our review provide a comprehensive evidence profile on the use of NSAIDs in ARTIs. Our results have been presented across different settings and populations to ensure convenience for practical reference by clinicians. Our review suggests that the current evidence supports the use of most NSAIDs with high‐certainty in fever control and with moderate certainty for sore throat. However, for ARDS‐related outcomes, mortality, duration, and course of the disease, the certainty of the evidence are low.

Our findings confirm that ibuprofen is an effective antipyretic in adults and children and may start acting in a shorter interval after administration compared to paracetamol. However, the evidence does not support that it can reduce fever more effectively than paracetamol. The effects of ibuprofen on cough differed substantially across trials; Probably because the pathophysiological pathways that generate cough are complicated and diverse, and ibuprofen affects them differently. Older studies suggested the use of ibuprofen for cough, but newer and higher‐quality evidence does not support its prescription for the sole purpose of relieving sore throat.

The evidence supports the use of naproxen for fever in adults, but its use for cough, sore throat, and more severe adverse outcomes like hospitalization, ARDS, and mortality is not supported by evidence. Naproxen prescription in children may be associated with an increased risk of gastrointestinal adverse events.

Diclofenac is also effective for fever reduction and may have a longer duration of effect compared with aspirin. However, its use for cough and sore throat cannot be suggested based on current evidence. Diclofenac has shown more common gastrointestinal adverse effects compared with celecoxib, and less common compared with aspirin. However, our confidence in these findings is low because of the low event rates in the underlying studies.

While the antipyretic effects of aspirin are confirmed in our systematic review, the evidence is uncertain regarding its precise effects on cough and sore throat and studies have conflicting results. It can also cause a small increase in the rate of gastrointestinal adverse effects.

Vaja et al^6^ published a systematic review and meta‐analysis on the safety of NSAIDs for usage in LRTIs, which examined mortality and the need for ventilation support. Their review that mainly consists of observational studies suggests a decline in mortality. In contrast, our review from 3 RCTs suggests no effect in either mortality or ventilator support for low‐dose aspirin. Salah et al^47^ in a recent meta‐analysis of aspirin efficacy in Covid‐19 also reported that aspirin did not affect mortality. Our findings suggest that ibuprofen may reduce mortality, however, our confidence in current evidence is low.

Vaja et al^6^ also concluded an uncertain result for ventilatory support from 1 RCT and 2 observational studies in their review. We found 3 RCTs all reporting little to no difference in the need for ventilatory support with an overall low certainty of evidence. The results of the International Severe Acute Respiratory and Emerging Infection Consortium^48^ study showed that prehospital NSAID use did not significantly affect symptoms severity, ICU admission, or ventilator needs among patients with Covid‐19.

Kim et al^3^ investigated the duration of disease, analgesic effect, and respiratory symptoms in a systematic review and meta‐analysis of NSAIDs in the common cold. Their meta‐analysis confirmed that NSAIDs effectively reduced pain in URTIs. Our results for diclofenac strongly suggest a large reduction in sore throat pain; however, the evidence for ibuprofen and naproxen is not as certain and shows a smaller magnitude of effect. Also, the evidence with moderate certainty shows that low‐dose aspirin did not affect sore throat pain.

In a meta‐analysis of 2 RCTs, Kim et al^3^ also concluded no reduction in coughing. Our review of 7 RCTs, with overall low confidence, showed that NSAIDs do not reduce cough. Winther and Mygind^24^ even observed an increase in coughing scores in patients who took ibuprofen with absent to mild cough.

Kim et al^3^ conducted a meta‐analysis on 2 RCTs and found no reduction in symptoms duration. Our results from 3 RCTs in inpatient settings confirmed the above finding in relatively sicker patients. However, in outpatient settings, 2 RCTs for ibuprofen and loxoprofen presented a small effect in reducing the duration of symptoms.

European and British health authorities have recommended the use of NSAIDs in the lowest dose and the shortest duration possible, considering concomitant drugs and conditions.^49^, ^50^, ^51^ Our meta‐analysis of high‐dose aspirin for 3 days suggests a higher incidence of gastrointestinal events compared with placebo; however, the results are not statistically significant (RR, 1.54; 95% CI, 0.92–2.60). For other NSAIDs, 9 RCTs in the adult ibuprofen group suggested no difference in results compared with placebo when used for a short duration. However, many children experienced gastrointestinal discomfort with ibuprofen in all 4 RCTs that included children.

Kim et al^3^ in 2015 conducted a Cochrane review on the overall efficacy of NSAIDs in upper URTIs; however, they did not compare the efficacy of different NSAIDs and did not review their efficacy in LRTIs. Also, Voiroit et al^52^ systematically reviewed the safety and complications of prehospital use of NSAIDs in patients with LRTI. Regardless of viral etiology, they strongly recommended against the prehospital use of NSAIDs; however, their recommendation is mostly based on evidence from observational data. Vaja et al^6^ also reviewed the safety of NSAIDs in adults with LRTIs—especially the ibuprofen controversy— and recommended that data be interpreted with caution because of poor quality.

Although NSAIDs are commonly prescribed in acute respiratory tract infections, the evidence supporting their safety and efficacy is limited. Our findings based on our comprehensive synthesis of available evidence show that NSAIDs seem to be beneficial in the outpatient management of fever and sore throat in adults and children. However, NSAIDs do not seem to decrease mortality or improve oxygenation in inpatient settings. We recommend more well‐designed RCTs in inpatient settings on patients with varying degrees of symptom severity, with long follow‐ups and systematic evaluations of any potential complications. Given the number of different medications as NSAIDs, a network meta‐analysis might also help synthesize the available evidence if studies with comparable outcome measures are available in the future.

### 4.1 Limitations

The wide scope of this review and use of nonhomogeneous variables by included studies prevented pooling of the data and meta‐analysis. The results of a systematic review are limited by the quality of included studies. Some of the included studies had significant methodological flaws, which can decrease our confidence in the results of this systematic review. Particularly, small sample sizes and low event rates were key concerns in many of the included studies.

RCTs that exclusively investigate the efficacy of naproxen, diclofenac, and celecoxib in viral respiratory infections are limited, and thus we need further robust studies to reach a consensus on their efficacy.

## 5 CONCLUSION

In conclusion, NSAIDs seem to be beneficial in the outpatient management of fever and sore throat in adults and children. However, they do not seem to decrease mortality or improve oxygenation in inpatient settings. Further RCTs with robust methodology and a larger sample size are recommended.

## ETHICS APPROVAL STATEMENT

Ethics code: IR.IUMS.FMD.REC.1399.507.

## DISCLOSURE

The authors declare no conflict of interest.

## AUTHORSHIP

R.V.A. and F.B. designed the search strategy. N.A., N.M.G., F.B., and P.P. screened, assessed, and extracted data from the articles. N.A. and Y.M. performed the meta‐analysis. N.A., F.B., H.R.B., and M.R.D. performed the review, wrote, and revised the manuscript. N.A. prepared the tables and figures. We confirm that the work is entirely that of the authors and that we alone satisfy the criteria for authorship.

## PERMISSION TO REPRODUCE MATERIAL FROM OTHER SOURCES

All used tools, applications, and gadgets are accordingly referenced in the manuscript.

### OPEN RESEARCH BADGES

This article has earned Open Data, Open Materials and Preregistered Research Design badges. Data, materials and the preregistered design and analysis plan are available at: https://ethics.research.ac.ir/PortalProposalListEn.php?code=IR.IUMS.FMD.REC.1399.507&title=&name=&stat=&isAll=&GlobalBackPage=https%3A%2F%2Fethics.research.ac.ir%2FPortalProposalList.php%3Fcode%3DIR.IUMS.FMD.REC.1399.507%26title%3D%26name%3D%26stat%3D%26isAll%3D%26GlobalBackPage%3Dhttps%253A%252F%252Fwww.google.com%252F.

## Data Availability

The data that support the findings of this study are openly available in the PubMed, Scopus, Web of Science, Cochrane, and Embase databases.

## APPENDIX 1

### March 18th 2020

#### PUBMED

1. ("Anti‐Inflammatory Agents, Non‐Steroidal"[Mesh] OR NSAID*[tiab] OR nonsteroid antiinflammatory agent*[tiab] OR "Indomethacin"[Mesh] OR Indomethacin*[tiab] OR "Diclofenac"[Mesh] OR "Diclofenac"[tiab] OR "Ibuprofen"[Mesh] OR Ibuprofen*[tiab] OR "Aspirin"[Mesh] OR Aspirin[tiab] OR Acetylsalicylic Acid*[tiab] OR "Naproxen"[Mesh] OR Naproxen*[tiab] OR "Ketorolac"[Mesh] OR Ketorolac*[tiab])
2. ("Coronavirus Infections"[Mesh] OR Coronavirus*[tiab] OR "COVID‐19"[Supplementary Concept] OR "COVID 19"[tiab] OR "severe acute respiratory syndrome coronavirus 2"[Supplementary Concept] OR "Respiratory Tract Infections"[Mesh] OR Respiratory Tract Infection*[tiab] OR Viral Infection*[tiab] OR "Virus Diseases"[Mesh] OR Virus Disease*[tiab] OR "Coronaviridae"[Mesh] OR Coronavirid*[tiab] OR "Pneumonia"[Mesh] OR Pneumonia*[tiab] OR Pneumonitis*[tiab] OR "Respiratory Distress Syndrome, Adult"[Mesh] OR Respiratory Distress Syndrome*[tiab] OR "ARDS" [tiab])
3. ("Clinical Trials as Topic"[Mesh] OR "Clinical Studies as Topic"[Mesh] OR "Randomized Controlled Trials as Topic"[Mesh] OR "In Vitro Techniques"[Mesh] OR "Clinical Trial"[pt] OR "Controlled Clinical Trial"[pt] OR "Randomized Controlled Trial"[pt] OR "Observational Study"[pt] OR "Observational Study, Veterinary"[pt] OR clinical trial*[tiab] OR controlled trial*[tiab] OR Clinical Stud*[tiab] OR Observational Stud*[tiab] OR "RCT"[tiab] OR in Vitro[tiab] OR Interventional study[tiab] OR Experimental Study[tiab])
4. #1 AND #2 AND #3 = 1058
  ("Anti‐Inflammatory Agents, Non‐Steroidal"[Mesh] OR NSAID*[tiab] OR nonsteroid antiinflammatory agent*[tiab] OR "Indomethacin"[Mesh] OR Indomethacin*[tiab] OR "Diclofenac"[Mesh] OR "Diclofenac"[tiab] OR "Ibuprofen"[Mesh] OR Ibuprofen*[tiab] OR "Aspirin"[Mesh] OR Aspirin[tiab] OR Acetylsalicylic Acid*[tiab] OR "Naproxen"[Mesh] OR Naproxen*[tiab] OR "Ketorolac"[Mesh] OR Ketorolac*[tiab]) AND ("Coronavirus Infections"[Mesh] OR Coronavirus*[tiab] OR "COVID‐19"[Supplementary Concept] OR "COVID 19"[tiab] OR "severe acute respiratory syndrome coronavirus 2"[Supplementary Concept] OR "Respiratory Tract Infections"[Mesh] OR Respiratory Tract Infection*[tiab] OR Viral Infection*[tiab] OR "Virus Diseases"[Mesh] OR Virus Disease*[tiab] OR "Coronaviridae"[Mesh] OR Coronavirid*[tiab] OR "Pneumonia"[Mesh] OR Pneumonia*[tiab] OR Pneumonitis*[tiab] OR "Respiratory Distress Syndrome, Adult"[Mesh] OR Respiratory Distress Syndrome*[tiab] OR "ARDS"[tiab]) AND ("Clinical Trials as Topic"[Mesh] OR "Clinical Studies as Topic"[Mesh] OR "Randomized Controlled Trials as Topic"[Mesh] OR "In Vitro Techniques"[Mesh] OR "Clinical Trial"[pt] OR "Controlled Clinical Trial"[pt] OR "Randomized Controlled Trial"[pt] OR "Observational Study"[pt] OR "Observational Study, Veterinary"[pt] OR clinical trial*[tiab] OR controlled trial*[tiab] OR Clinical Stud*[tiab] OR Observational Stud*[tiab] OR "RCT"[tiab] OR in Vitro[tiab] OR Interventional study[tiab] OR Experimental Study[tiab])

#### EMBASE

1. (“nonsteroid antiinflammatory agent”/syn OR “NSAID*”:ti,ab OR “Indomethacin”/syn OR “indomethacin*”:ti,ab OR “diclofenac”/syn OR “diclofenac”:ti,ab OR “ibuprofen”/syn OR “ibuprofen*”:ti,ab OR “acetylsalicylic acid”/syn OR “acetylsalicylic acid*”:ti,ab OR “aspirin”:ti,ab OR “naproxen”/syn or “naproxen*”:ti,ab OR “ketorolac”/syn OR “ketorolac*”:ti,ab)
2. (“Coronavirus infection”/syn OR “Coronavirus*”:ti,ab OR “covid 19”:ti,ab OR “respiratory tract infection”/syn OR “respiratory tract infection*”:ti,ab OR “virus pneumonia”/syn OR “virus pneumonia”:ti,ab OR “viral infection*”:ti,ab OR “virus disease*”:ti,ab OR “Coronaviridae”/syn OR “Coronavirid*”:ti,ab OR “pneumonia”/syn OR Pneumonia*:ti,ab OR Pneumonitis*:ti,ab OR “adult respiratory distress syndrome”/syn OR “respiratory distress syndrome”:ti,ab OR “ARDS”:ti,ab)
3. (“clinical trial (topic)”/syn OR “clinical study”/syn OR “controlled clinical trial”/syn OR “controlled clinical trial (topic)”/syn OR “randomized controlled trial (topic)”/syn OR “clinical trial*”:ti,ab OR “controlled trial*”:ti,ab OR “clinical stud*”:ti,ab OR “RCT”:ti,ab)
4. #1 AND #2 AND #3 = 22726
  (“nonsteroid antiinflammatory agent”/syn OR “NSAID*”:ti,ab OR “Indomethacin”/syn OR “indomethacin*”:ti,ab OR “diclofenac”/syn OR “diclofenac”:ti,ab OR “ibuprofen”/syn OR “ibuprofen*”:ti,ab OR “acetylsalicylic acid”/syn OR “acetylsalicylic acid*”:ti,ab OR “aspirin”:ti,ab OR “naproxen”/syn or “naproxen*”:ti,ab OR “ketorolac”/syn OR “ketorolac*”:ti,ab) AND (“Coronavirus infection”/syn OR “Coronavirus*”:ti,ab OR “covid 19”:ti,ab OR “respiratory tract infection”/syn OR “respiratory tract infection*”:ti,ab OR “virus pneumonia”/syn OR “virus pneumonia”:ti,ab OR “viral infection*”:ti,ab OR “virus disease*”:ti,ab OR “Coronaviridae”/syn OR “Coronavirid*”:ti,ab OR “pneumonia”/syn OR Pneumonia*:ti,ab OR Pneumonitis*:ti,ab OR “adult respiratory distress syndrome”/syn OR “respiratory distress syndrome”:ti,ab OR “ARDS”:ti,ab) AND (“clinical trial (topic)”/syn OR “clinical study”/syn OR “controlled clinical trial”/syn OR “controlled clinical trial (topic)”/syn OR “randomized controlled trial (topic)”/syn OR “clinical trial*”:ti,ab OR “controlled trial*”:ti,ab OR “clinical stud*”:ti,ab OR “RCT”:ti,ab)

#### SCOPUS

1. (TITLE‐ABS‐KEY ( nonsteroid* antiinflammatory W/3 agent* ) OR TITLE‐ABS‐KEY ( NSAID* OR Indomet*acin* OR Osmosin OR Indocid OR Metindol OR Amuno Or Indocin OR Diclo*enac* OR Dicrofenac OR Dichlofenal OR Diclinate OR Feloran OR Voltarol OR Novapirina OR Ort*o*en OR Voltaren OR Ibuprofen* OR Ibumetin OR Motrin OR Nuprin OR Rufen OR "Salprofen OR Trauma Dolgit Gel" OR Brufen OR Aspirin OR Acylpyrin OR Aloxiprimum OR Colfarit OR Dispril OR Easprin OR Ecotrin OR Endosprin OR Magnecyl OR Micristin OR Polopirin OR Polopiryna OR Solprin OR Solupsan OR Zorprin OR Acetysal OR Acetylsalicylic Acid* OR Naproxen* OR Methoxypropiocin OR Anaprox OR "Aleve" OR "Proxen" OR Synflex OR Naprosin OR Naprosyn OR Ketorolac* ))
2. (TITLE‐ABS‐KEY ( Coronavirus* OR "Covid 19" OR "Respiratory Tract Infection*" OR "Viral Infection*" OR "Virus Disease*" OR Coronavirid* OR Pneumonia* OR Pneumonitis* OR "Respiratory Distress Syndrome" OR "ARDS" ))
3. (TITLE‐ABS‐KEY ( "Clinical Trial*" OR "Randomi?ed Controlled Trial*" OR "RCT" OR "Clinical Stud*" OR "Observation* Stud*" OR "In vitro" OR "Intervention* Stud*" OR "Experiment* Stud*" ))
4. #1 AND #2 AND #3 = 3567
  (TITLE‐ABS‐KEY ( nonsteroid* antiinflammatory W/3 agent* ) OR TITLE‐ABS‐KEY ( NSAID* OR Indomet*acin* OR Osmosin OR Indocid OR Metindol OR Amuno Or Indocin OR Diclo*enac* OR Dicrofenac OR Dichlofenal OR Diclinate OR Feloran OR Voltarol OR Novapirina OR Ort*o*en OR Voltaren OR Ibuprofen* OR Ibumetin OR Motrin OR Nuprin OR Rufen OR "Salprofen OR Trauma Dolgit Gel" OR Brufen OR Aspirin OR Acylpyrin OR Aloxiprimum OR Colfarit OR Dispril OR Easprin OR Ecotrin OR Endosprin OR Magnecyl OR Micristin OR Polopirin OR Polopiryna OR Solprin OR Solupsan OR Zorprin OR Acetysal OR Acetylsalicylic Acid* OR Naproxen* OR Methoxypropiocin OR Anaprox OR "Aleve" OR "Proxen" OR Synflex OR Naprosin OR Naprosyn OR Ketorolac* )) AND (TITLE‐ABS‐KEY ( Coronavirus* OR "Covid 19" OR "Respiratory Tract Infection*" OR "Viral Infection*" OR "Virus Disease*" OR Coronavirid* OR Pneumonia* OR Pneumonitis* OR "Respiratory Distress Syndrome" OR "ARDS" )) AND (TITLE‐ABS‐KEY ("Clinical Trial*" OR "Randomi?ed Controlled Trial*" OR "RCT" OR "Clinical Stud*" OR "Observation* Stud*" OR "In vitro" OR "Intervention* Stud*" OR "Experiment* Stud*" ))

#### WEB OF SCIENCE

1. (TS=(nonsteroid* antiinflammatory NEAR/3 agent*) OR TS=(NSAID* OR Indomet*acin* OR Osmosin OR Indocid OR Metindol OR Amuno OR Indocin OR Diclo*enac* OR Dicrofenac OR Dichlofenal OR Diclinate OR Feloran OR Voltarol OR Novapirina OR Ortofen OR Orthofen OR Orthophen OR Voltaren OR Ibuprofen* OR Ibumetin OR Motrin OR Nuprin OR Rufen OR Salprofen OR "Trauma Dolgit Gel" OR Brufen OR Aspirin OR Acylpyrin OR Aloxiprimum OR Colfarit OR Dispril OR Easprin OR Ecotrin OR Endosprin OR Magnecyl OR Micristin OR Polopirin OR Polopiryna OR Solprin OR Solupsan OR Zorprin OR Acetysal OR Acetylsalicylic Acid* OR Naproxen* OR Methoxypropiocin OR Anaprox OR "Aleve" OR "Proxen" OR Synflex OR Naprosin OR Naprosyn OR Ketorolac*))
2. (TS=(Coronavirus* OR "Covid 19" OR "Respiratory Tract Infection*" OR "Viral Infection*" OR "Virus Disease*" OR Coronavirid* OR Pneumonia* OR Pneumonitis* OR "Respiratory Distress Syndrome" OR "ARDS"))
3. (TS=("Clinical Trial*" OR "Randomi?ed Controlled Trial*" OR "RCT" OR "Clinical Stud*" OR "Observation* Stud*" OR "In vitro" OR "Intervention* Stud*" OR "Experiment* Stud*"))
4. #1 AND #2 AND #3 = 228
  (TS=(nonsteroid* antiinflammatory NEAR/3 agent*) OR TS=(NSAID* OR Indomet*acin* OR Osmosin OR Indocid OR Metindol OR Amuno OR Indocin OR Diclo*enac* OR Dicrofenac OR Dichlofenal OR Diclinate OR Feloran OR Voltarol OR Novapirina OR Ortofen OR Orthofen OR Orthophen OR Voltaren OR Ibuprofen* OR Ibumetin OR Motrin OR Nuprin OR Rufen OR Salprofen OR "Trauma Dolgit Gel" OR Brufen OR Aspirin OR Acylpyrin OR Aloxiprimum OR Colfarit OR Dispril OR Easprin OR Ecotrin OR Endosprin OR Magnecyl OR Micristin OR Polopirin OR Polopiryna OR Solprin OR Solupsan OR Zorprin OR Acetysal OR Acetylsalicylic Acid* OR Naproxen* OR Methoxypropiocin OR Anaprox OR "Aleve" OR "Proxen" OR Synflex OR Naprosin OR Naprosyn OR Ketorolac*)) AND (TS=(Coronavirus* OR "Covid 19" OR "Respiratory Tract Infection*" OR "Viral Infection*" OR "Virus Disease*" OR Coronavirid* OR Pneumonia* OR Pneumonitis* OR "Respiratory Distress Syndrome" OR "ARDS")) AND (TS=("Clinical Trial*" OR "Randomi?ed Controlled Trial*" OR "RCT" OR "Clinical Stud*" OR "Observation* Stud*" OR "In vitro" OR "Intervention* Stud*" OR "Experiment* Stud*"))

#### COCHRANE LIBRARY

1. ([mh "Anti‐Inflammatory Agents, Non‐Steroidal"] OR NSAID*:ti,ab OR nonsteroid antiinflammatory NEAR/3 agent*:ti,ab OR [mh "Indomethacin"] OR Indomethacin*:ti,ab OR [mh "Diclofenac"] OR Diclofenac*:ti,ab OR [mh "Ibuprofen"] OR Ibuprofen*:ti,ab OR [mh "Aspirin"] OR Aspirin:ti,ab OR "Acetylsalicylic Acid*":ti,ab OR [mh "Naproxen"] OR Naproxen*:ti,ab OR [mh "Ketorolac"] OR Ketorolac*:ti,ab)
2. ([mh "Coronavirus Infections"] OR Coronavirus*:ti,ab OR "COVID 19":ti,ab OR [mh "Respiratory Tract Infections"] OR "Respiratory Tract Infection*":ti,ab OR "Viral Infection*":ti,ab OR [mh "Virus Diseases"] OR "Virus Disease*":ti,ab OR [mh "Coronaviridae"] OR Coronavirid*:ti,ab OR [mh "Pneumonia"] OR Pneumonia*:ti,ab OR Pneumonitis*:ti,ab OR [mh "Respiratory Distress Syndrome, Adult"] OR "Respiratory Distress Syndrome*":ti,ab OR "ARDS":ti,ab)
3. ([mh "Clinical Trials as Topic"] OR [mh "Clinical Studies as Topic"] OR [mh "Randomized Controlled Trials as Topic"] OR [mh "In Vitro Techniques"] OR "Clinical Trial":pt OR "Controlled Clinical Trial":pt OR "Randomized Controlled Trial":pt OR "Observational Study":pt OR "clinical trial*":ti,ab OR "controlled trial*":ti,ab OR Clinical Stud*:ti,ab OR "Observational Stud*":ti,ab OR "RCT":ti,ab OR "in Vitro":ti,ab OR Interventional study:ti,ab OR Experimental Study:ti,ab)
4. #1 AND #2 AND #3 = 288 (281 Trial and 7 Cochrane Reviews)
  ([mh "Anti‐Inflammatory Agents, Non‐Steroidal"] OR NSAID*:ti,ab OR nonsteroid antiinflammatory NEAR/3 agent*:ti,ab OR [mh "Indomethacin"] OR Indomethacin*:ti,ab OR [mh "Diclofenac"] OR Diclofenac*:ti,ab OR [mh "Ibuprofen"] OR Ibuprofen*:ti,ab OR [mh "Aspirin"] OR Aspirin:ti,ab OR "Acetylsalicylic Acid*":ti,ab OR [mh "Naproxen"] OR Naproxen*:ti,ab OR [mh "Ketorolac"] OR Ketorolac*:ti,ab) AND ([mh "Coronavirus Infections"] OR Coronavirus*:ti,ab OR "COVID 19":ti,ab OR [mh "Respiratory Tract Infections"] OR "Respiratory Tract Infection*":ti,ab OR "Viral Infection*":ti,ab OR [mh "Virus Diseases"] OR "Virus Disease*":ti,ab OR [mh "Coronaviridae"] OR Coronavirid*:ti,ab OR [mh "Pneumonia"] OR Pneumonia*:ti,ab OR Pneumonitis*:ti,ab OR [mh "Respiratory Distress Syndrome, Adult"] OR "Respiratory Distress Syndrome*":ti,ab OR "ARDS":ti,ab) AND ([mh "Clinical Trials as Topic"] OR [mh "Clinical Studies as Topic"] OR [mh "Randomized Controlled Trials as Topic"] OR [mh "In Vitro Techniques"] OR "Clinical Trial":pt OR "Controlled Clinical Trial":pt OR "Randomized Controlled Trial":pt OR "Observational Study":pt OR "clinical trial*":ti,ab OR "controlled trial*":ti,ab OR Clinical Stud*:ti,ab OR "Observational Stud*":ti,ab OR "RCT":ti,ab OR "in Vitro":ti,ab OR Interventional study:ti,ab OR Experimental Study:ti,ab)

### NOVEMBER 11TH 2020

#### PUBMED

("Anti‐Inflammatory Agents, Non‐Steroidal"[Mesh] OR NSAID*[tiab] OR nonsteroid antiinflammatory agent*[tiab] OR "Indomethacin"[Mesh] OR Indomethacin*[tiab] OR "Diclofenac"[Mesh] OR "Diclofenac"[tiab] OR "Ibuprofen"[Mesh] OR Ibuprofen*[tiab] OR "Aspirin"[Mesh] OR Aspirin[tiab] OR Acetylsalicylic Acid*[tiab] OR "Naproxen"[Mesh] OR Naproxen*[tiab] OR "Ketorolac"[Mesh] OR Ketorolac*[tiab]) AND ("Coronavirus Infections"[Mesh] OR Coronavirus*[tiab] OR "COVID‐19"[Supplementary Concept] OR "COVID 19"[tiab] OR "severe acute respiratory syndrome coronavirus 2"[Supplementary Concept] OR "Respiratory Tract Infections"[Mesh] OR Respiratory Tract Infection*[tiab] OR Viral Infection*[tiab] OR "Virus Diseases"[Mesh] OR Virus Disease*[tiab] OR "Coronaviridae"[Mesh] OR Coronavirid*[tiab] OR "Pneumonia"[Mesh] OR Pneumonia*[tiab] OR Pneumonitis*[tiab] OR "Respiratory Distress Syndrome, Adult"[Mesh] OR Respiratory Distress Syndrome*[tiab] OR "ARDS"[tiab]) AND ("Clinical Trials as Topic"[Mesh] OR "Clinical Studies as Topic"[Mesh] OR "Randomized Controlled Trials as Topic"[Mesh] OR "In Vitro Techniques"[Mesh] OR "Clinical Trial"[pt] OR "Controlled Clinical Trial"[pt] OR "Randomized Controlled Trial"[pt] OR "Observational Study"[pt] OR "Observational Study, Veterinary"[pt] OR clinical trial*[tiab] OR controlled trial*[tiab] OR Clinical Stud*[tiab] OR Observational Stud*[tiab] OR "RCT"[tiab] OR in Vitro[tiab] OR Interventional study[tiab] OR Experimental Study[tiab]) AND ("2020/01/01"[dp] : "2020/11/10"[dp]) = 62

#### EMBASE

(“nonsteroid antiinflammatory agent”/syn OR “nonsteroid antiinflammatory agent” OR “nsaid*”:ti,ab OR “indomethacin”/syn OR “indomethacin” OR “indomethacin*”:ti,ab OR “diclofenac”/syn OR “diclofenac” OR “diclofenac”:ti,ab OR “ibuprofen”/syn OR “ibuprofen” OR “ibuprofen*”:ti,ab OR “acetylsalicylic acid”/syn OR “acetylsalicylic acid” OR “acetylsalicylic acid*”:ti,ab OR “aspirin”:ti,ab OR “naproxen”/syn OR “naproxen” OR “naproxen*”:ti,ab OR “ketorolac”/syn OR “ketorolac” OR “ketorolac*”:ti,ab) AND (“coronavirus infection”/syn OR “coronavirus infection” OR “coronavirus*”:ti,ab OR “covid 19”:ti,ab OR “respiratory tract infection”/syn OR “respiratory tract infection” OR “respiratory tract infection*”:ti,ab OR “virus pneumonia”/syn OR “virus pneumonia” OR “virus pneumonia”:ti,ab OR “viral infection*”:ti,ab OR “virus disease*”:ti,ab OR “coronaviridae”/syn OR “coronaviridae” OR “coronavirid*”:ti,ab OR “pneumonia”/syn OR “pneumonia” OR pneumonia*:ti,ab OR pneumonitis*:ti,ab OR “adult respiratory distress syndrome”/syn OR “adult respiratory distress syndrome” OR “respiratory distress syndrome”:ti,ab OR “ards”:ti,ab) AND (“clinical trial (topic)”/syn OR “clinical trial (topic)” OR “clinical study”/syn OR “clinical study” OR “controlled clinical trial”/syn OR “controlled clinical trial” OR “controlled clinical trial (topic)”/syn OR “controlled clinical trial (topic)” OR “randomized controlled trial (topic)”/syn OR “randomized controlled trial (topic)” OR “clinical trial*”:ti,ab OR “controlled trial*”:ti,ab OR “clinical stud*”:ti,ab OR “rct”:ti,ab) AND [clinical study]/lim AND [humans]/lim AND ([1‐1‐2020]/sd NOT [10‐11‐2020]/sd) = 2211

#### SCOPUS

(TITLE‐ABS‐KEY ( nonsteroid* AND antiinflammatory W/3 agent* ) OR TITLE‐ABS‐KEY ( nsaid* OR indomet*acin* OR osmosin OR indocid OR metindol OR amuno OR indocin OR diclo*enac* OR dicrofenac OR dichlofenal OR diclinate OR feloran OR voltarol OR novapirina OR ort*o*en OR voltaren OR ibuprofen* OR ibumetin OR motrin OR nuprin OR rufen OR "Salprofen OR Trauma Dolgit Gel" OR brufen OR aspirin OR acylpyrin OR aloxiprimum OR colfarit OR dispril OR easprin OR ecotrin OR endosprin OR magnecyl OR micristin OR polopirin OR polopiryna OR solprin OR solupsan OR zorprin OR acetysal OR acetylsalicylic AND acid* OR naproxen* OR methoxypropiocin OR anaprox OR "Aleve" OR "Proxen" OR synflex OR naprosin OR naprosyn OR ketorolac* ) ) AND ( TITLE‐ABS‐KEY ( coronavirus* OR "Covid 19" OR "Respiratory Tract Infection*" OR "Viral Infection*" OR "Virus Disease*" OR coronavirid* OR pneumonia* OR pneumonitis* OR "Respiratory Distress Syndrome" OR "ARDS" ) ) AND ( TITLE‐ABS‐KEY ( "Clinical Trial*" OR "Randomi?ed Controlled Trial*" OR "RCT" OR "Clinical Stud*" OR "Observation* Stud*" OR "In vitro" OR "Intervention* Stud*" OR "Experiment* Stud*" ) ) AND ( LIMIT‐TO ( PUBYEAR, 2020 ) ) = 237

#### WEB OF SCIENCE

(TS=(nonsteroid* antiinflammatory NEAR/3 agent*) OR TS=(NSAID* OR Indomet*acin* OR Osmosin OR Indocid OR Metindol OR Amuno OR Indocin OR Diclo*enac* OR Dicrofenac OR Dichlofenal OR Diclinate OR Feloran OR Voltarol OR Novapirina OR Ortofen OR Orthofen OR Orthophen OR Voltaren OR Ibuprofen* OR Ibumetin OR Motrin OR Nuprin OR Rufen OR Salprofen OR "Trauma Dolgit Gel" OR Brufen OR Aspirin OR Acylpyrin OR Aloxiprimum OR Colfarit OR Dispril OR Easprin OR Ecotrin OR Endosprin OR Magnecyl OR Micristin OR Polopirin OR Polopiryna OR Solprin OR Solupsan OR Zorprin OR Acetysal OR Acetylsalicylic Acid* OR Naproxen* OR Methoxypropiocin OR Anaprox OR "Aleve" OR "Proxen" OR Synflex OR Naprosin OR Naprosyn OR Ketorolac*)) AND (TS=(Coronavirus* OR "Covid 19" OR "Respiratory Tract Infection*" OR "Viral Infection*" OR "Virus Disease*" OR Coronavirid* OR Pneumonia* OR Pneumonitis* OR "Respiratory Distress Syndrome" OR "ARDS")) AND (TS=("Clinical Trial*" OR "Randomi?ed Controlled Trial*" OR "RCT" OR "Clinical Stud*" OR "Observation* Stud*" OR "In vitro" OR "Intervention* Stud*" OR "Experiment* Stud*")) AND PY=(2020) = 34

#### COCHRANE LIBRARY

1. #1 AND #2 AND #3 = 288 (281 Trials and 7 Cochrane Reviews) – 521 (510 Trials and 11 Cochrane Reviews)
  ([mh "Anti‐Inflammatory Agents, Non‐Steroidal"] OR NSAID*:ti,ab OR nonsteroid antiinflammatory NEAR/3 agent*:ti,ab OR [mh "Indomethacin"] OR Indomethacin*:ti,ab OR [mh "Diclofenac"] OR Diclofenac*:ti,ab OR [mh "Ibuprofen"] OR Ibuprofen*:ti,ab OR [mh "Aspirin"] OR Aspirin:ti,ab OR "Acetylsalicylic Acid*":ti,ab OR [mh "Naproxen"] OR Naproxen*:ti,ab OR [mh "Ketorolac"] OR Ketorolac*:ti,ab) AND ([mh "Coronavirus Infections"] OR Coronavirus*:ti,ab OR "COVID 19":ti,ab OR [mh "Respiratory Tract Infections"] OR "Respiratory Tract Infection*":ti,ab OR "Viral Infection*":ti,ab OR [mh "Virus Diseases"] OR "Virus Disease*":ti,ab OR [mh "Coronaviridae"] OR Coronavirid*:ti,ab OR [mh "Pneumonia"] OR Pneumonia*:ti,ab OR Pneumonitis*:ti,ab OR [mh "Respiratory Distress Syndrome, Adult"] OR "Respiratory Distress Syndrome*":ti,ab OR "ARDS":ti,ab) AND ([mh "Clinical Trials as Topic"] OR [mh "Clinical Studies as Topic"] OR [mh "Randomized Controlled Trials as Topic"] OR [mh "In Vitro Techniques"] OR "Clinical Trial":pt OR "Controlled Clinical Trial":pt OR "Randomized Controlled Trial":pt OR "Observational Study":pt OR "clinical trial*":ti,ab OR "controlled trial*":ti,ab OR Clinical Stud*:ti,ab OR "Observational Stud*":ti,ab OR "RCT":ti,ab OR "in Vitro":ti,ab OR Interventional study:ti,ab OR Experimental Study:ti,ab)
